# Predicting long-term mortality in spontaneous intracerebral hemorrhage patients using the advanced lung cancer inflammation index

**DOI:** 10.3389/fneur.2025.1610341

**Published:** 2025-09-24

**Authors:** Wanqiu Lv, Xiahui Li, Yangchun Xiao, Fang Fang, Yi Xu, Yu Zhang

**Affiliations:** ^1^School of Basic Medical Sciences and School of Nursing, Chengdu University, Chengdu, China; ^2^Department of Neurosurgery, Clinical Medical College and Affiliated Hospital of Chengdu University, Chengdu, China; ^3^Department of Neurosurgery, West China Hospital, Sichuan University, Chengdu, China

**Keywords:** spontaneous intracerebral hemorrhage, advanced lung cancer inflammation index, long-term mortality, prognosis, ALI

## Abstract

**Background:**

The Advanced Lung Cancer Inflammation Index (ALI), a composite measure of inflammatory and nutritional status, has demonstrated prognostic value across various diseases. Given the involvement of systemic inflammation and malnutrition in post-stroke pathophysiology, ALI may serve as a physiologically relevant indicator for predicting long-term outcomes in patients with spontaneous intracerebral hemorrhage (ICH). This study aimed to assess the predictive accuracy of ALI for long-term mortality in ICH patients.

**Methods:**

This retrospective cohort study, conducted at West China Hospital, Sichuan University, included 2,541 patients with spontaneous ICH. Neutrophil count, lymphocyte count, serum albumin, and BMI were recorded within 24 h of admission to calculate ALI patients were into quartiles based on ALI values. Cox proportional hazards regression and restricted cubic spline analyses were used to evaluate the association between ALI and long-term mortality.

**Results:**

RCS analyses demonstrated a linear association between higher ALI values and a lower risk of all-cause mortality. This inverse relationship remained consistent after excluding in-hospital deaths, underscoring the robustness of the findings. Patients with higher ALI values, indicating better inflammatory and nutritional status, exhibited improved long-term survival. Furthermore, ROC analysis showed that ALI had modestly better predictive performance compared to other biomarkers (AUC = 0.67), highlighting its potential clinical utility in outcome prediction and management.

**Conclusion:**

In patients with spontaneous ICH, ALI is a practical and independent prognostic indicator of long-term mortality. By integration inflammatory and nutritional factors, ALI enhances risk stratification and may support individualized clinical decision-making.

## 1 Introduction

Spontaneous ICH is a severe neurological condition that account for approximately 10–20% of all acute strokes and is associated with significant mortality and disability (1). Long-term outcomes after ICH remain poor, with 10-year mortality rates ranging from 16.7% in younger patients to as high as 90.0% in older adults (2). In China, the 1-month case fatality rate is around 12.5%, and the 5-year mortality rate can reach 22.2–53% (3). Globally, long-term mortality following ICH has been reported to exceed 40–50%, underscoring its persistent burden (4). Although recent studies have shown a decline in short-term case fatality rates in some regions-likely due to advances in acute care and medical interventions (5, 6)- the long-term outcomes for ICH patients remain a cause for concern. Their mortality rates are substantially higher than those of the general population, despite these improvements. While much of the existing research has focused on predicting short-term mortality, there is a critical gap in our understanding of the long-term prognosis of ICH survivors, particularly in terms of functional recovery and post-stroke complications (7). The factors that influence long-term mortality in ICH are complex and not fully understood. Systemic inflammation, nutritional status, and comorbidities all potentially contributing to poor outcomes (7, 8). Thus, identifying reliable prognostic markers is essential for improving long-term care and guiding therapeutic decisions for ICH patients.

Emerging clinical and experimental evidence underscores the pivotal role of inflammation in determining outcomes following ICH (8). Previous studies have highlighted the close association between stroke outcomes and markers of nutritional status and systemic inflammation, such as serum albumin levels and body weight (9, 10). These factors affect not only the severity of neurological injury but also recovery potential, highlighting the importance of integration inflammation and nutritional indicators into prognostic models (9).

The ALI index, a composite marker that combines serum albumin concentration, BMI, and the NLR, offers a novel approach to assessing systemic inflammation and nutritional status (11). Originally developed as a prognostic tool in oncology, ALI has shown promise across various inflammation-related conditions, including stroke, heart failure, hypertension, and coronary artery disease (12–14). Its application in acute neurological conditions such as ICH is particularly noteworthy, as it provides a holistic assessment of systemic physiological status-both inflammation and nutritional- which are critical determinants of clinical outcomes.

This study aims to evaluate the prognostic value of ALI for long-term mortality in patients with spontaneous ICH. Additionally, we assess its potential to support clinical decision-making, improve outcome prediction, and enhance risk stratification in high-risk populations. In addition to biological determinants, early process-of-care factors such as stroke unit admission, blood pressure management, and surgical intervention have also been shown to significantly influence long-term outcomes in ICH patients. Thus, identifying reliable prognostic markers is essential for improving long-term care and guiding therapeutic decisions for ICH patients (15).

## 2 Methods and subjects

### 2.1 Study design and data source

From December 2010 to August 2019, 2,541 patients with spontaneous ICH were identified retrospectively from West China Hospital’s computerized medical records at Sichuan University. The People’s Republic of China’s National Death Registry provided the survival data, guaranteeing comprehensive mortality statistics for all participant. Ethical approval was granted by the West China Hospital Review Board (Approval Number: 2022–705). This study adhered to the STROBE reporting guidelines and was approved by the ethics committee in accordance with the Declaration of Helsinki.

### 2.2 Patient selection

The investigation included all adults (≥ 18 years old) who had a spontaneous ICH diagnosis confirmed by brain MRI or CT at admission and subsequently evaluated by a neurologist. Inclusion criteria required a definite diagnosis of spontaneous ICH and complete baseline clinical data, including serum albumin, lymphocyte count, neutrophil count, BMI, and NLR, all measured within 24 h of hospitalization.

Patient exclusion criteria were as follows: (1) hemorrhagic transformation of ischemic stroke, brain trauma, subarachnoid hemorrhage, and hemorrhagic vascular malformation; (2) missing or invalid 24-h baseline data, such as BMI, serum albumin, lymphocyte count, neutrophil count, or NLR; and (3) incomplete or missing mortality data in the national registry. Incomplete information or records of deceased patients stored in the family record management system. The aim of this study was to compile a robust dataset to explore the predictive value of ALI in determining the long-term outcomes of patients with ICH.

### 2.3 Laboratory data collection and clinical characteristics

Demographic data, including age, gender, and lifestyle habits such as smoking and alcohol use, were collected. Additionally, medical history, encompassing conditions like hypertension and diabetes mellitus, alongside clinical parameters documented during hospitalization, was analyzed. Hypertension was defined as a known diagnosis, use of antihypertensive medications, or systolic blood pressure ≥140 mmHg at admission. Diabetes mellitus was defined as a known diagnosis, use of insulin or oral hypoglycemic agents, or fasting blood glucose ≥7.0 mmol/L. The ABC / 2 formula was employed to calculate hematoma volume and assess location (supratentorial or infratentorial). Systolic blood pressure was measured at admission. The Glasgow Coma Scale (GCS) score was categorized into two groups: GCS ≤ 8 and GCS > 8 the presence of intraventricular hemorrhage was also recorded. For patients who underwent craniotomy, laboratory values including neutrophil count, lymphocyte count, and serum albumin level were obtained from the first blood sample collected within 24 h of admission.

### 2.4 Definition and logarithmic transformation of ALI

ALI is a composite index that combines markers of inflammation and nutritional status to provide a more comprehensive assessment of a patient’s systemic health. It is calculated by dividing the product of the serum albumin level and BMI by the NLR (12). The ALI was initially classified into quartiles based on its distribution within the cohort for the objectives of this study: Quartile 1 (0.588–5.327), Quartile 2 (5.328–8.937), Quartile 3 (8.948–15.257), and Quartile 4 (15.258–40.459). The examination of the correlation between ALI and long-term mortality across a wide range of values was facilitated by this quartile-based categorization.

Additionally, to simplify interpretation and further assess the prognostic utility of ALI, it was also dichotomized based on the cohort median. This generated two groups: a low ALI group (ALI < 20.52) and a high ALI group (ALI ≥ 20.52). Subgroup analyses were conducted to compare long-term mortality between these two groups.

### 2.5 Outcomes

Long-term mortality, which was defined as all-cause mortality during the longest available follow-up period, was the primary outcome. The secondary outcome focused on long-term mortality among patients who were discharged alive. Follow-up mortality data were sourced from the Chinese household registration system, recognized for its high accuracy and reliability in maintaining death records.

### 2.6 Statistical analysis

Statistical analyses were conducted using R software (version 4.2.2) and MSTATA. The baseline characteristics of the study population were summarized using descriptive statistics. Group comparisons were performed using the Student’s t-test when appropriate. Continuous variables were reported as either mean ± SD or median with IQR, depending on the distribution of the data. Categorical variables, presented as frequencies and percentages, were compared using chi-square tests or Fisher’s exact tests, as appropriate. Kaplan–Meier survival curves were employed to estimate survival probabilities across different ALI quartiles.

Cox proportional hazards regression models, adjusted for confounders such as age, GCS, hematoma volume, diabetes, and the supratentorial or infratentorial location of hematomas, were developed to assess the association between ALI and long-term mortality. Subgroup analyses were performed to evaluate effect modification by stratifying patients based on variables such as age, sex, and hematoma location.

In order to investigate potential dose–response relationships between ALI and long-term mortality, RCS models were implemented. By incorporating four percentile-based knots of the ALI distribution, these models facilitated a detailed investigation of non-linear associations. To explore the relationship between ALI and prognosis, ALI values were categorized into quartiles and also dichotomized at the median. This approach enabled an exploratory assessment of risk gradients and allowed comparison of survival outcomes across ALI strata. While these data-driven thresholds do not necessarily correspond to clinically validated cut-offs, they provide initial insight into ALI’s discriminative capacity.

## 3 Results

### 3.1 Baseline characteristics and group comparisons

**Figure 1:**
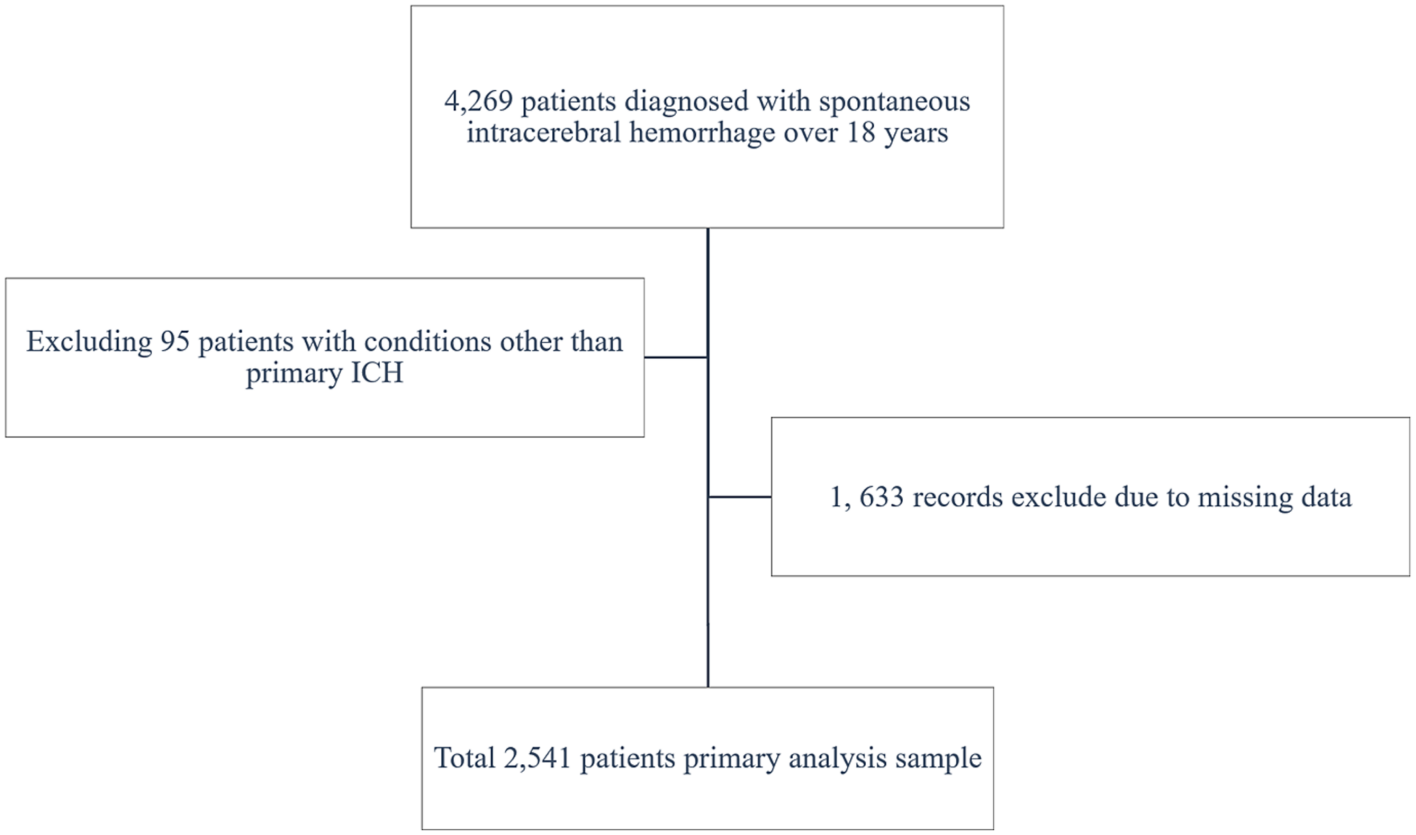
Flow diagram for the selection of participants included in the present analysis.

A total of 2,541 patients with spontaneous ICH were included in the study (shown in Figure 1). The patients’ baseline includes are displayed (shown in Table 1), grouped by ALI quartiles. The cohort’s median age was 56.61 ± 14.53 years. Significant differences were observed across ALI quartiles for variables such as age, GCS score, SBP, and hematoma size. Patients in higher ALI quartiles (Quartiles 3 and 4) were more likely to have better GCS scores and smaller hematoma volumes, suggesting that patients with higher ALI values were generally in better clinical condition at baseline.

**Table 1:** Baseline characteristics of the patients.

Characteristics	Overal (2,541)	Advanced lung cancer inflammation index				*p*
		0.588–5.327 (635)	5.328–8.937 (635)	8.948–15.257 (635)	15.258–40.459 (636)	
Demographic						
Age, years, Mean ± SD	56.51 ± 14.53	58.848 ± 15.16	56.89 ± 14.28	56.48 ± 14.15	54.27 ± 14.25	<0.01
Female	817(32.15)	217 (33.86)	202 (31.81)	208 (32.76)	192 (30.19)	0.55
Smoking, *n* (%)						
Never	1872 (73.67)	490 (77.17)	467 (73.54)	474 (67.24)	441(69.81)	
Yes	669 (26.33)	145 (22.83)	168 (26.46)	161 (25.35)	195 (30.66)	<0.01
Alcohol, *n* (%)	801 (31.52)	175 (27.56)	209 (32.91)	189 (29.76)	228 (35.85)	<0.01
Hypertension, *n* (%)	1853 (72.92)	485 (76.38)	467 (73.54)	473 (74.49)	428 (67.30)	<0.01
Diabetes, *n* (%)	252 (10.16)	59 (9.29)	70 (11.02)	63 (9.92)	65 (10.22)	0.78
Hematoma characteristics						
Hematoma size	24.33 ± 28.58	32.29 ± 30.92	26.29 ± 27.11	23.57 ± 32.61	15.2 ± 19.12	<0.01
Hematoma infratentorial, *n* (%)	544 (21.41)	120 (18.90)	148 (23.31)	140 (22.05)	136 (21.38)	0.27
Hematoma intraventricular, *n* (%)	635 (24.99)	198 (31.18)	180 (28.35)	143 (22.52)	114 (17.92)	<0.01
GCS, Mean ± SD	10.28 ± 4.22	8.45 ± 3.93	9.45 ± 4.2	10.76 ± 4	12.76 ± 3.61	<0.01
SBP, Mean ± SD	161.18 ± 32.56	165.2 ± 33.76	163.51 ± 33.44	161.94 ± 31.57	154.08 ± 30.32	<0.01
Neutrophil-to-lymphocyte ratio, Mean ± SD	12.72 ± 9.77	24.69 ± 11.53	13.26 ± 3.47	8.39 ± 2.36	4.54 ± 1.56	<0.01
Lymphocyte, Mean ± SD	1.01 ± 0.57	0.55 ± 0.23	0.87 ± 0.53	1.11 ± 0.39	1.52 ± 0.55	<0.01
Albumin, Mean ± SD	3.81 ± 0.65	3.58 ± 0.72	3.82 ± 0.66	3.87 ± 0.6	3.99 ± 0.51	<0.01
Neutrophil, Mean ± SD	9.82 ± 5.39	12.38 ± 4.7	11.2 ± 7.39	9.08 ± 3.61	6.63 ± 2.7	<0.01

Mean ± SD; *n* (%), “Hematoma infratentorial” and “Hematoma intraventricular” describe the hematoma location, GCS refers to the Glasgow Coma Scale, and p-values represent the statistical significance of differences between groups.

### 3.2 Survival analysis

**Figure 2:**
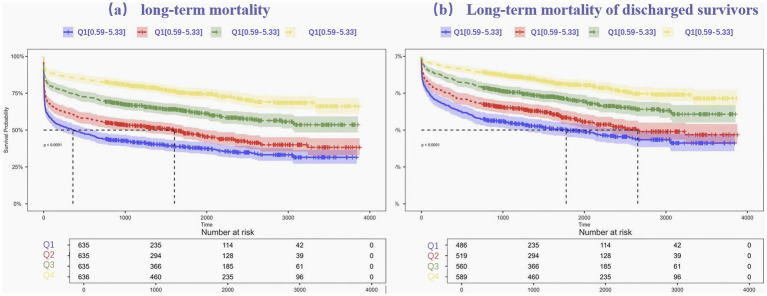
Kaplan–Meier estimates for long-term mortality **(a)** and Long-term mortality of discharged survivors **(b)** for patients with intracerebral hemorrhage.

To evaluate long-term mortality across different ALI stages, Kaplan–Meier survival analysis was applied. Survival outcomes for the entire cohort, categorized into ALI quartiles (see Figure 2), revealed that individuals in Quartile 4 demonstrated markedly better long-term survival compared to those in Quartile 1, with the log-rank test showing statistical significance (*p* < 0.001). The prognostic value of ALI in predicting long-term survival was highlighted by the finding that mortality was highest in Quartile 1 and lowest in Quartile 4.

Kaplan–Meier survival curves were further generated to further investigate the association between ALI and long-term mortality, after in-hospital fatalities were excluded. The survival differences among ALI quartiles remained statistically significant, with Quartile 4 continuing to show the most favorable survival outcomes. The log-rank test for this subset analysis also yielded a *p*-value < 0.001, reinforcing the role of ALI as a reliable predictor of long-term mortality.

### 3.3 Cox proportional hazards regression

In order to examine the relationship between long-term mortality and ALI in patients with spontaneous ICH, Cox proportional hazards regression models were implemented. These models were adjusted for potential covariates, including age, smoking and alcohol consumption, diabetes, systolic blood pressure, hematoma volume, the presence of infratentorial or intraventricular hematomas, and GCS score, as detailed shown in Table 2.

**Table 2:** The associations between quartile of ALI and long-term mortality in all patients and Long-term mortality of discharged survivors using multivariate Cox regression.

Outcome	ALI Level	Unadjusted HR (95% CI)	*p*	Multivariable Regression Adjusted HR (95% CI)	*p*
Long-term mortality	Continuous variable	94 (0.93–0.94)	<0.001	0.97 (0.96–0.98)	<0.001
	Dichotomous variable*	0.34 (0.27–0.43)	<0.001	0.62 (0.49–0.79)	<0.001
	0.588–5.327	Ref		Ref	
	5.328–8.937	0.76 (0.66–0.88)	< 0.001	0.92 (0.79–1.06)	0.250
	8.948–15.257	0.48 (0.41–0.56)	< 0.001	0.71 (0.60–0.83)	<0.001
	15.258–40.459	0.29 (0.24–0.35)	< 0.001	0.60 (0.50–0.73)	<0.001
Long-term mortality of discharged survivors	Continuous variable	0.94 (0.93–0.95)	<0.001	0.97 (0.96–0.98)	< 0.001
	Dichotomous variable*	0.37 (0.28–0.48)	<0.001	0.61 (0.47–0.81)	<0.001
	0.588–5.327	Ref		Ref	
	5.328–8.937	0.76 (0.64–0.91)	0.003	0.90 (0.75–1.09)	0.285
	8.948–15.257	0.48 (0.40–0.58)	< 0.001	0.66 (0.54–0.81)	<0.001
	15.258–40.459	0.29 (0.23–0.36)	< 0.001	0.51 (0.42–0.68)	<0.001

*indicates that the dichotomous variable of ALI was derived using the best cutoff value determined by ROC analysis with the Youden Index, which balances sensitivity and specificity. This cutoff was used to stratify patients into two groups for further analysis.

Two distinct models were constructed to examine this association. Model 1 evaluated the association between ALI and long-term mortality, including deaths occurring during hospitalization. Model 2 specifically analyzed post-discharge outcomes by excluding in-hospital deaths to reduce the influence of acute-phase confounding and better isolate the prognostic relevance of ALI for long-term survivors. In Model 1, individuals in Quartile 4 (the highest ALI quartile) showed a substantially reduced risk of long-term mortality compared to those in Quartile 1 (the lowest quartile), with an adjusted HR of 0.60 (95% CI, 0.50–0.73, *p* < 0.001). Similarly, Model 2 confirmed this relationship after excluding in-hospital deaths. The risk of long-term mortality remained significantly lower in Quartile 4 than in Quartile 1, with an adjusted HR of 0.51 (95% CI, 0.42–0.68, *p* < 0.001).

To maintain the clinical interpretability raw ALI values were also used in supplementary analyses, including restricted cubic spline models and Kaplan–Meier survival curves. These results further support the effectiveness of ALI as a predictive marker for assessing long-term mortality risk in ICH patients.

### 3.4 Predictive performance of ALI and other inflammatory markers

**Figure 3:**
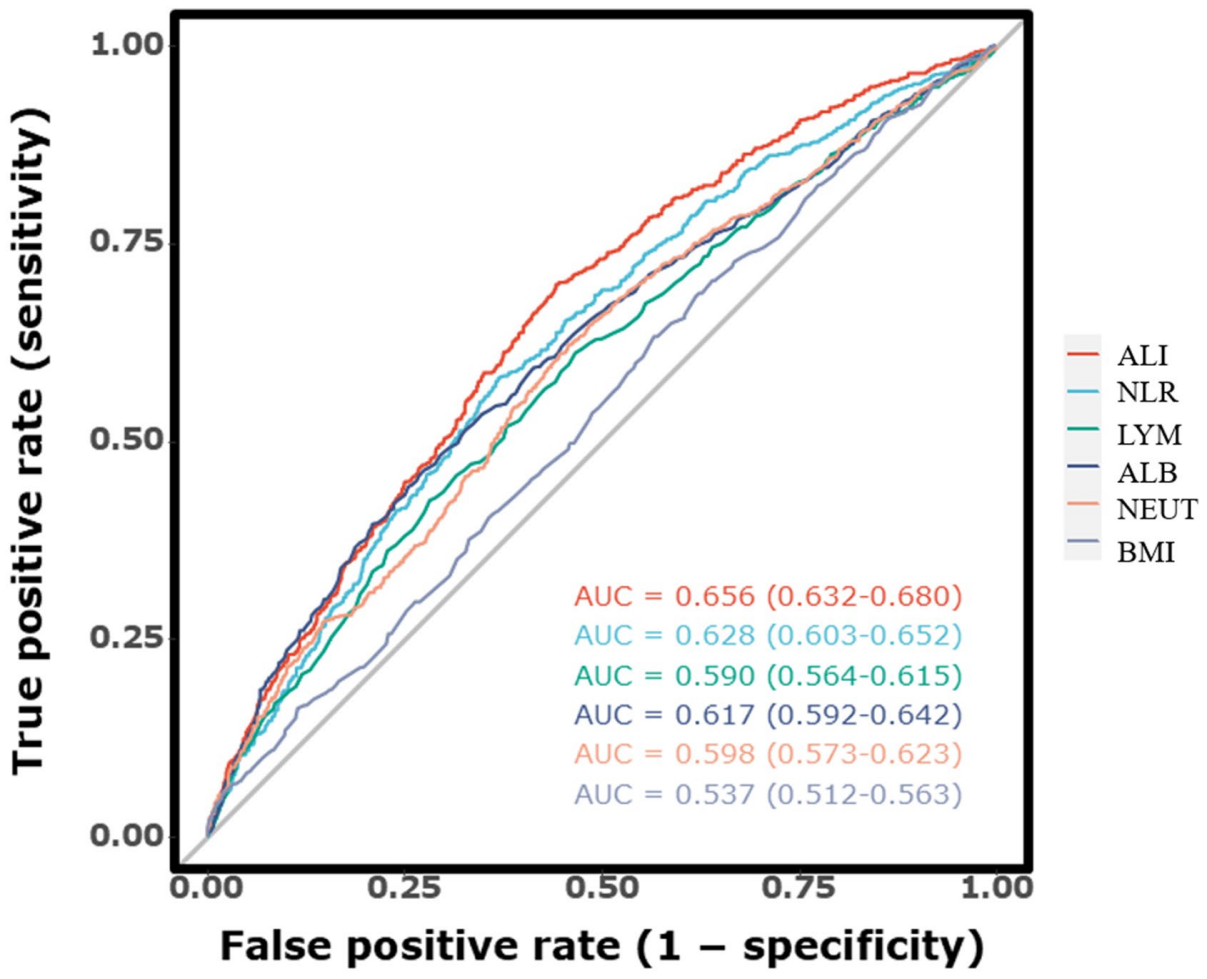
The receiver operating characteristic curves illustrating the predictive value of the ALI for long-term mortality.

The discriminative ability of ALI and other inflammatory markers for predicting long-term mortality was evaluated using ROC curves, as illustrated in Figure 3. The AUC for ALI was 0.672 (95% CI, 0.651–0.693), indicating strong predictive accuracy for long-term mortality in ICH patients. In comparison, the AUC for NLR was slightly lower at 0.640 (95% CI, 0.619–0.662), suggesting that ALI is a more reliable predictor. Furthermore, the AUC values for individual indicators including lymphocyte count, neutrophil count, BMI, and serum albumin were also analyzed. The respective AUC values were lymphocyte count (0.587, 95% CI, 0.564–0.609), neutrophil count (0.617, 95% CI, 0.595–0.639), BMI (0.538, 95% CI, 0.516–0.561), and serum albumin (0.628, 95% CI, 0.606–0.650). Among all markers, ALI achieved the highest AUC, underscoring its modestly better predictive capability.

### 3.5 Restricted cubic spline analysis

**Figure 4:**
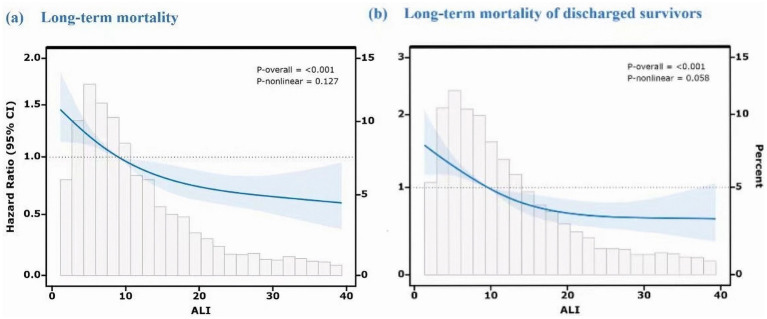
Dose–response relationship between ALI and long-term mortality using restricted cubic spline analysis. Both models were adjusted for age, hematoma volume, hematoma location (supratentorial/infratentorial), GCS score, diabetes, and other potential confounders. The Y-axis represents the hazard ratio (HR) relative to the reference ALI value (median, 50th percentile). **(a)** Long-term mortality (including in-hospital deaths). **(b)** Long-term mortality of discharged survivors (excluding in-hospital deaths).

In order to examine the dose–response relationship between ALI and long-term mortality, an RCS analysis was implemented. In the initial model (shown in Figure 4a), which encompassed all fatalities, low ALI values were associated with a higher mortality risk, which plateaued at the median and decreased as ALI increased. This suggests that better survival rates are possible with improved inflammatory and nutritional status. The second model (shown in Figure 4b), excluding in-hospital deaths, showed a more linear decline in mortality risk with higher ALI, indicating its stronger prognostic value for post-discharge outcomes.

This research underscores the significance of ALI as a valuable indicator for the identification of high-risk patients and the customization of interventions to improve long-term survival periods.

### 3.6 Subgroup analyses

**Figure 5:**
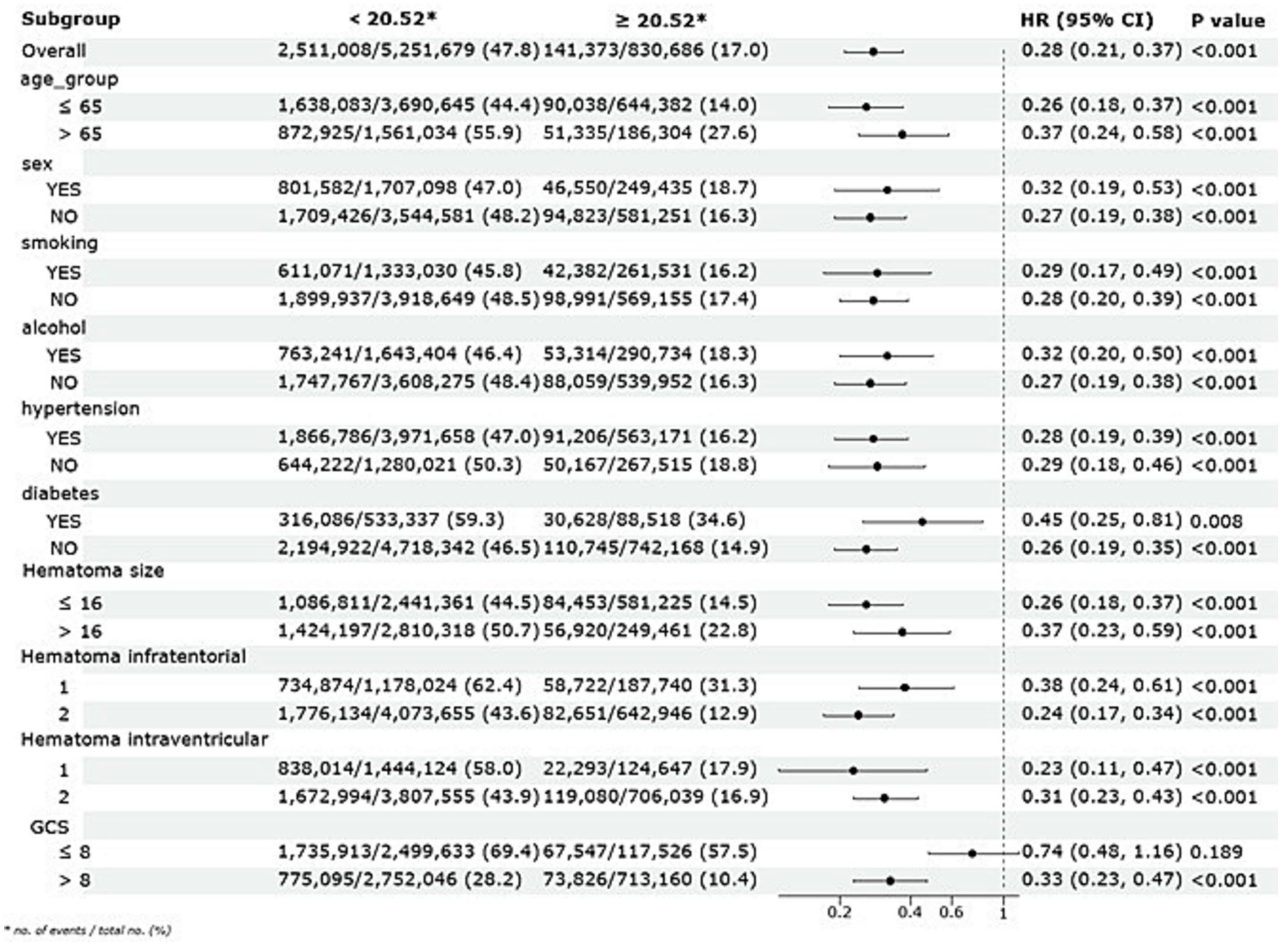
Subgroup analysis of the association between ALI and long-term mortality with a multivariate Cox regression model.

Subgroup analyses were conducted to evaluate the stability of the association between ALI and long-term mortality across various demographic and clinical factors, including age, sex, smoking and alcohol consumption, hypertension, diabetes, and hematoma size, as illustrated in Figure 5. The results consistently demonstrated a protective effect of higher ALI quartiles across all subgroups. Patients—whether young or old, male or female, smokers or non-smokers, drinkers or non-drinkers, with or without hypertension or diabetes, and across various hematoma sizes—showed significantly improved long-term survival in the higher ALI quartiles, with log-rank *p*-values below 0.05 in all comparisons. Furthermore, interaction p-values exceeding 0.05 indicated no significant interactions between ALI and any subgroup variable.

## 4 Discussion

The ALI demonstrates modestly better predictive ability for long-term mortality compared to conventional markers such as the NLR. Unlike the NLR, which reflects immune balance (16), the ALI integrates both inflammatory and nutritional components and provides a comprehensive assessment of patient health (17). This integration likely strengthens ALI’s prognostic value in ICH, where both inflammation and nutrition impact outcomes (18). In the acute phase of ICH, excessive neutrophil infiltration leads to the release of reactive oxygen species (ROS), matrix metalloproteinases (e.g., MMP-9), and pro-inflammatory cytokines (e.g., IL-1β, TNF-α), disrupting the blood–brain barrier, exacerbating edema, and promoting neuronal injury (19, 20). Simultaneously, hypoalbuminemia and low BMI, which reflect impaired nutritional reserves, are associated with reduced plasma oncotic pressure, worsened vasogenic edema, and diminished endogenous antioxidant and anti-inflammatory defense (9, 10). Thus, ALI captures both the inflammatory injury burden and the patient’s physiological capacity to recover. ALI’s simplicity and cost-effectiveness make it a valuable tool for improving clinical prognostic accuracy in ICH.

The relationship between ALI and mortality can be attributed to its capacity to reflect both the severity of the inflammatory response and the patient’s overall resilience to stress (20, 21). Similar to findings in cancer patients, elevated ALI levels in ICH patients may indicate an imbalance between systemic inflammation and nutritional reserves, potentially impairing recovery and leading to higher mortality (22). Malnutrition, which is common in critically ill patients, exacerbates outcomes among various diseases, including stroke (23). By integration both inflammatory and nutritional markers, ALI offers a more comprehensive assessment of patient health, which is particularly crucial in ICH, where recovery is influenced by both the initial hemorrhage and the patient’s overall health status (24).

The limitations of the study must be considered, despite the promising results. First, the retrospective nature of the analysis inherently restricts the capacity to establish a causal relationship between ALI and long-term mortality. We are unable to exclude the possibility of residual disturbances as a result of unmeasured or incompletely documented factors, such as changes in post-discharge treatment or rehabilitation, despite the fact that we have adjusted for key disturbance variables (25). Second, while ALI provides a robust measure integration systemic inflammation and nutritional status, it does not encompass certain pathophysiological nuances specific to ICH, such as the dynamics of hematoma evolution or localized inflammatory responses. Additionally, the reliance on a single measurement of ALI at admission may not fully capture its temporal variations, which could offer further insights into the trajectory of recovery or clinical deterioration. Third, our study did not include direct comparisons between ALI and established prognostic scoring systems for ICH, such as the ICH Score or FUNC Score. These models are widely used in clinical practice, and future research should assess whether incorporating ALI can enhance their predictive accuracy.

Although this study benefits from a large and well-defined cohort, its findings are based on data from a single-center population. This limitation may affect the applicability of the results to broader or more diverse patient groups with varying clinical practices and healthcare systems. While subgroup and spline analyses were conducted to explore consistency within the cohort, formal internal validation such as bootstrapping or cross-validation was not performed, this may limit the generalizability of the predictive associations observed. Future research should therefore incorporate there validation techniques and expand to multicenter settings to strengthen external validity. Additionally, efforts should be made to refine the integration of ALI into comprehensive risk assessment frameworks, aiming to support tailored management approaches for patients with intracerebral hemorrhage. Although ALI was categorized using median and quartile thresholds for exploratory analysis, future studies should aim to identify clinically meaningful cut-off points based on outcome-driven methods such as ROC or decision curve analysis.

In summary, our investigation underscores the potential of ALI as a practical and reliable prognostic indicator of long-term mortality in ICH. As a simple and accessible biomarker, ALI offers clinicians a valuable tool for early risk stratification and decision-making mortality in ICH. By capturing both inflammatory and nutritional dimensions, ALI provides a more holistic assessment of patient status than conventional inflammatory makers. Further multicenter, prospective studies are warranted to validate our findings and facilitate the integration of ALI into clinical practice.

## Data Availability

The original contributions presented in the study are included in the article/Supplementary material, further inquiries can be directed to the corresponding authors.
