# Supplementary material for: Predicting long-term mortality in spontaneous intracerebral hemorrhage patients using the advanced lung cancer inflammation index
Source: Front Neurol. 2025 Sep 24;16:1610341. doi: 10.3389/fneur.2025.1610341 (PMC12509695; doi:10.3389/fneur.2025.1610341)
Supplement: Supplementary file 1 [file Table_1.DOCX]

Table S1 Univariate and multivariate analysis of influencing factors (Cox regression)

| Characteristic | Univariable | | | | | | Multivariable | | | |  |
| --- | --- | --- | --- | --- | --- | --- | --- | --- | --- | --- | --- |
|  | N | HR^1^ | 95% CI^1^ | p-value | N | HR^1^ | | 95% CI^1^ | p-value | | |
| Demographics |  |  |  |  |  |  | |  |  | | |
| Age, years, mean (SD) | 2,541 | 1.02 | 1.02, 1.02 | <0.001 | 2,541 | 1.02 | | 1.02, 1.02 | <0.001 | | |
| Female, n (%) | 817 | 0.99 | 0.88, 1.12 | 0.901 | Na | Na | | Na | Na | | |
| smoking, n (%) | 669 | 0.86 | 0.75, 0.98 | 0.025 | 669 | 0.94 | | 0.80, 1.10 | 0.408 | | |
| alcohol, n (%) | 801 | 0.89 | 0.79, 1.01 | 0.080 | 801 | 1.02 | | 0.88, 1.19 | 0.756 | | |
| hypertension, n (%) | 1,853 | 1.02 | 0.89, 1.16 | 0.801 | Na | Na | | Na | Na | | |
| diabetes, n (%) | 257 | 1.37 | 1.15, 1.63 | <0.001 | 257 | 1.11 | | 0.93, 1.33 | 0.231 | | |
| GCS, mean (SD) | 2,541 | 0.83 | 0.81, 0.84 | <0.001 | 2,541 | 0.85 | | 0.83, 0.86 | <0.001 | | |
| SBP, mean (SD) | 2,541 | 1.00 | 1.00, 1.00 | 0.762 | Na | Na | | Na | Na | | |
| Hematoma characteristics |  |  |  |  |  |  | |  |  | | |
| Hematoma size, cm, mean (SD) | 2,541 | 1.01 | 1.00, 1.01 | <0.001 | 2,541 | 1.00 | | 1.00, 1.00 | 0.001 | | |
| Hematoma infratentorial, n (%) | 1,997 | 0.62 | 0.55, 0.71 | <0.001 | 1,997 | 0.71 | | 0.62, 0.81 | <0.001 | | |
| Hematoma intraventricular, n (%) | 1,906 | 0.67 | 0.59, 0.76 | <0.001 | 1,906 | 0.87 | | 0.76, 0.99 | 0.031 | | |
| ALI |  |  |  |  |  |  | |  |  | | |
| 1 | 635 | — | — |  | 635 | — | | — |  | | |
| 2 | 635 | 0.76 | 0.66, 0.88 | <0.001 | 635 | 0.92 | | 0.80, 1.07 | 0.300 | | |
| 3 | 635 | 0.48 | 0.41, 0.56 | <0.001 | 635 | 0.72 | | 0.61, 0.84 | <0.001 | | |
| 4 | 636 | 0.29 | 0.24, 0.35 | <0.001 | 636 | 0.62 | | 0.51, 0.75 | <0.001 | | |
| ^1^HR = Hazard Ratio, CI = Confidence Interval; Na: not available; | | | | | | | | | |  |  |
| SBP: systolic blood pressure; GCS: Glasgow Coma Scale; | | | | | | | | | |  |  |
